# Molecular Regulation of Porcine Skeletal Muscle Development: Insights from Research on CDC23 Expression and Function

**DOI:** 10.3390/ijms25073664

**Published:** 2024-03-25

**Authors:** Su Xie, Quan Liu, Chong Fu, Yansen Chen, Mengxun Li, Cheng Tian, Jiaxuan Li, Min Han, Changchun Li

**Affiliations:** 1 Key Laboratory of Swine Genetics and Breeding of the Ministry of Agriculture, College of Animal Science and Technology, Huazhong Agricultural University, Wuhan 430070, China; xiesu@webmail.hzau.edu.cn (S.X.); liuquan.hzau.edu.cn@webmail.hzau.edu.cn (Q.L.);; 2 TERRA Teaching and Research Center, University of Liège, Gembloux Agro-Bio Tech (ULiège-GxABT), 5030 Gembloux, Belgium; yansen.chen@uliege.be

**Keywords:** CDC23, porcine satellite cells (PSCs), myoblast differentiation, GSEA, myoblast proliferation, cell cycle pathway

## Abstract

Cell division cycle 23 (CDC23) is a component of the tetratricopeptide repeat (TPR) subunit in the anaphase-promoting complex or cyclosome (APC/C) complex, which participates in the regulation of mitosis in eukaryotes. However, the regulatory model and mechanism by which the CDC23 gene regulates muscle production in pigs are largely unknown. In this study, we investigated the expression of CDC23 in pigs, and the results indicated that CDC23 is widely expressed in various tissues and organs. In vitro cell experiments have demonstrated that CDC23 promotes the proliferation of myoblasts, as well as significantly positively regulating the differentiation of skeletal muscle satellite cells. In addition, Gene Set Enrichment Analysis (GSEA) revealed a significant downregulation of the cell cycle pathway during the differentiation process of skeletal muscle satellite cells. The protein–protein interaction (PPI) network showed a high degree of interaction between genes related to the cell cycle pathway and CDC23. Subsequently, in differentiated myocytes induced after overexpression of CDC23, the level of CDC23 exhibited a significant negative correlation with the expression of key factors in the cell cycle pathway, suggesting that CDC23 may be involved in the inhibition of the cell cycle signaling pathway in order to promote the differentiation process. In summary, we preliminarily determined the function of CDC23 with the aim of providing new insights into molecular regulation during porcine skeletal muscle development.

## 1. Introduction

Skeletal muscle accounts for about 40% of total body weight [1], making it the largest metabolic-endocrine organ, and plays a vital role in protein storage, metabolism, and the maintenance of body homeostasis [2]. Mammalian skeletal muscle development is a complex stepwise process that includes the following three stages: firstly, the somites develop into progenitor cells, followed by myoblast proliferation, migration, and fusion, before the final differentiation into fast- or slow-twitch muscle fibers which can distinguish different types of muscles [3]. Inhibition of myoblast differentiation impairs muscle formation and regeneration [4,5,6]. Therefore, it is important to elucidate the regulatory mechanisms of myoblast differentiation in maintaining skeletal muscle mass and function.

Skeletal muscle development is a complex process regulated by various factor networks [7,8], such as signaling pathways [9], fibroblast growth factors [10], and insulin-like growth factors [11]. Many studies have reported that long non-coding RNAs (lncRNAs) can participate in skeletal muscle development and play an important role in the proliferation and differentiation process [12,13]. For example, lncRNA MEG3 overexpression may relieve the inhibitory effect on serum response factor (SRF) and myoblast differentiation induced by miR-423-5p [14]. H19 regulates PSC differentiation through direct binding with Drebrin 1 (DBN1) [15]. LncRNA TCONS_00323213 interacts with PBX/Knotted Homeobox 2 (PKNOX2) to promote the differentiation of PSC by relieving the inhibition of PKNOX2 on myogenin (MyoG) [16]. Many classical pathways play key roles in muscle proliferation and differentiation processes, such as the canonical Wnt signaling pathway [17,18], the Notch signaling pathway [19], and the mTOR signaling pathway [20]. In addition, some hormones, such as parathyroid hormone [21] and thyroid hormone [22], are also involved in regulating skeletal muscle development. The NFAT5 pathway participates in muscle development and regeneration by regulating the differentiation of myoblasts into multinucleated myotubes [23]. Furthermore, MEK5/ERK5 pathway activation by Yes-associated protein (YAP) during muscle cell differentiation has recently been reported [24]. Many proteins also play important roles in muscle differentiation, such as the MADS box protein Mef2 [25,26]. Fu et al. [27] have reported that Egl nine homolog 3 (EGLN3, also known as PHD3) regulates skeletal muscle differentiation by modulating the stability of the myogenin protein. Cell division control protein 42 homolog (Cdc42) has been identified as a negative regulator of the differentiation of skeletal muscle cells [28]. Similarly, the homeobox protein Hox-A11 has a significant inhibitory effect on myogenesis during muscle differentiation [29].

The proliferation of skeletal muscle satellite cells is closely associated with the positive regulation of the cell cycle. However, withdrawal of the cell cycle in myogenic cells and an increase in muscle-specific gene expression are prerequisites for myogenic differentiation [30,31,32]. During myogenesis, the activation of p38 MAPK promotes cell cycle exit by inducing the expression of a cyclin-dependent kinase inhibitor, p21, which facilitates terminal differentiation of muscle precursor cells [33,34]. Interestingly, myogenic differentiation (MyoD) can also regulate cell cycle arrest by inducing p21 (Cdkn1a) [35,36]. Furthermore, recent research findings have indicated that MyoD controls cell cycle exit and myogenic gene expression through its synergistic interaction with a master regulator of the cell cycle progression named Rb (the protein product of the retinoblastoma gene) [37]. The research conducted by Charasse et al. revealed the crucial role of RhoA activity regulation in myoblasts for cell cycle exit, skeletal muscle differentiation gene expression, and myotube fusion [38]. Therefore, it is of great significance to elucidate the functions of novel cell cycle regulatory factors and their regulatory networks in skeletal muscle differentiation and regeneration processes.

In our preliminary research, we conducted pulldown experiments and mass spectrometry analysis of MEG3 [39]. We noted the presence of a protein, CDC23, in the mass spectrometry results. Therefore, we speculate that CDC23 may be involved in the regulation of muscle development by MEG3. Then, we further conducted protein–protein interaction (PPI) network analysis using the STRING database. Through PPI analysis, we found that CDC23 is closely associated with significantly enriched genes in the cell cycle pathway.

In the current research, we found that the specific regulatory role and mechanism of CDC23 (especially the pig CDC23 gene) in muscle development remains poorly defined. In this study, we detected the expression profile of CDC23 and explored its function and molecular mechanism in the myogenesis of porcine satellite cells. Our data revealed a new regulatory network of CDC23 in skeletal muscle, which may contribute to a better understanding of the mechanism underlying the epigenetic regulation of skeletal muscle development and regeneration, and will accelerate the process of genetic improvement in animals.

## 2. Results

### 2.1. Significant Inhibition of Cell Cycle Pathways during Satellite Cell Differentiation

In our preliminary research, we constructed cDNA libraries from two differentiation time points (24 and 36 h), and 6341 DEGs were detected between the two groups after sequencing [15]. In order to elucidate the functions, signaling pathways, and upstream regulators of the functional gene sets involved in the process of differentiation, we conducted GO and KEGG biological process enrichment of the 6341 DEGs using Gene Set Enrichment Analysis (GSEA).

**Figure 1:**
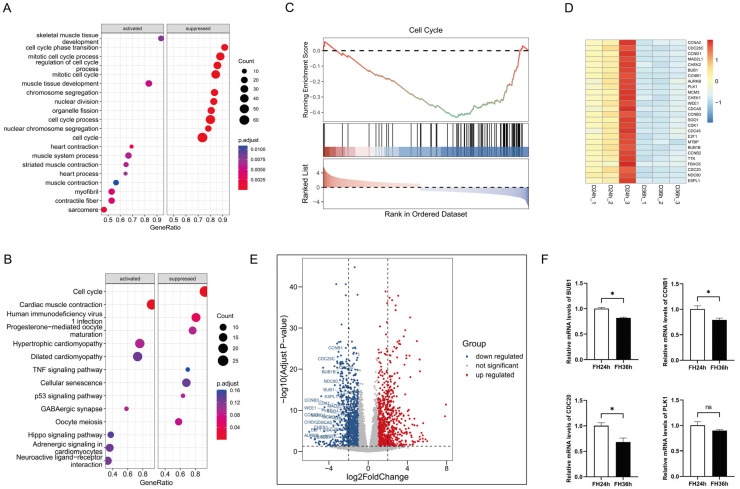
Functional annotation of DEGs in the differentiation process of skeletal muscle satellite cells by using GSEA. (**A**) Gene ontology analysis. (**B**) KEGG pathway analysis. (**C**) The top suppressed gene set in D36h was ranked by NES. Heatmap plots (**D**) and volcano plots (the dotted line in (**E**) indicates |log2FoldChange| = 2) (**E**) of 26 core enrichment genes in the cell cycle pathway. (**F**) Validation via RT-qPCR of several DE mRNAs from RNA-seq. (* *p* < 0.05). ns is considered to be not significant.

The GO term enrichment (Figure 1A) results revealed that the gene sets suppressed in the 36 h group relative to the 24 h group were primarily involved in cell cycle phase transition, the mitotic cell cycle process, regulation of the cell cycle process, the mitotic cell cycle, chromosome segregation, nuclear division, organelle fission, and the cell cycle process, whereas the activated gene sets were mainly involved in processes such as skeletal muscle tissue development, muscle tissue development, heart contraction, the muscle system process, striated muscle contraction, muscle contraction, myofibrils, and sarcomeres. We performed KEGG pathway analysis (Figure 1B) and the results indicated that, for DEGs in D36h, the top downregulated gene sets belonged to the cell cycle pathway, and the top upregulated gene sets belonged to cardiac muscle contraction.

We further verified the top enriched KEGG pathways using GSEA and discovered that 26 genes in the cell cycle pathway (Figure 1C), including CDC25C, CCNB1, CDC20, and CDCA5, were prominently downregulated in the 36 h group (Figure 1D,E). We then randomly selected several DEGs mentioned above and detected their expression in skeletal muscle satellite cells using real-time fluorescent quantitative PCR, and the data indicated that the RT-qPCR results were highly consistent with the RNA sequencing results, demonstrating the accuracy and reliability of our bioinformatics analysis results (Figure 1F).

### 2.2. Potential Role of CDC23 in Myoblast Differentiation

**Figure 2:**
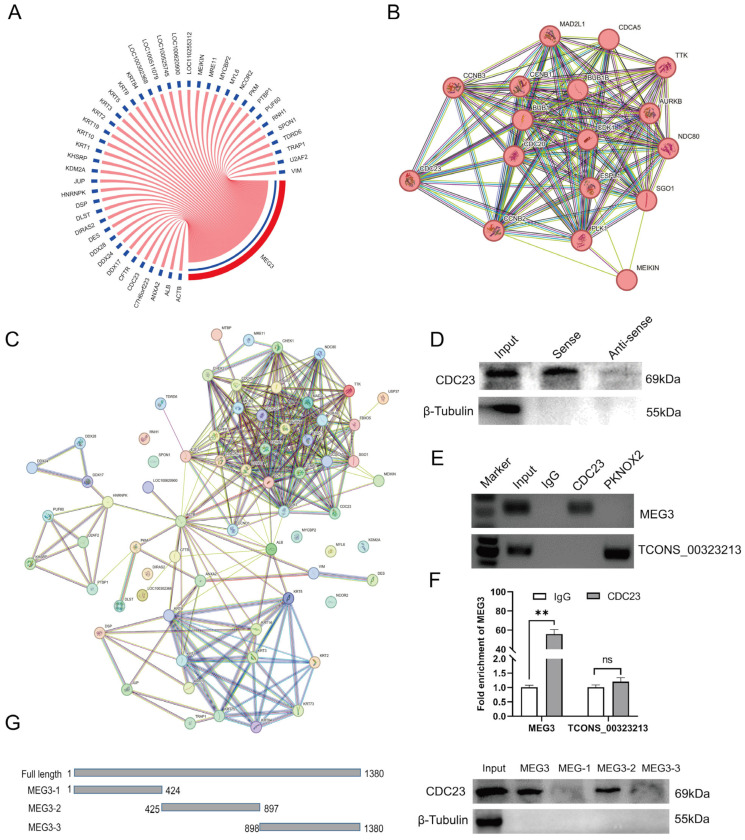
Identification of the CDC23 interaction relationships. (**A**) Circos plots of MEG3 and its 44 potential interaction proteins. (**B**,**C**) The protein–protein interactions between potential interacting proteins of MEG3 and core-enriched genes in the cell cycle pathway were retrieved using the STRING database. (**D**) Western blotting results showed that MEG3 specifically binds to the CDC23 protein. (**E**) RNA immunoprecipitation (RIP) results indicated that the CDC23 protein binds to MEG3. The PKNOX2 protein and lnc-TCONS_00323213 were used as the negative control. (**F**) RIP-RT-qPCR results indicated that MEG3 was significantly enriched by the CDC23 protein. (**G**) The interaction of truncated MEG3 and CDC23 was determined via RNA pull-down. Error bars are the mean ± standard error of the mean (SEM) of three biological replicates. Statistical differences between groups were determined with a 2-tailed Student’s *t*-test, and a *p*-value < 0.05 was considered statistically significant (** *p* < 0.01). ns is considered to be not significant.

The previous results showed that lncRNA-MEG3 regulated the cell cycle of skeletal muscle satellite cells, inhibited proliferation, and promoted differentiation [14]. By performing RNA-binding protein experiments (pull down) on lncRNA-MEG3 in vitro, as well as mass spectrometry analysis of the resulting RNA-protein complexes [39], we obtained a protein of interest, CDC23, with a size of 69 kDa. The mass spectrometry analysis results are shown in Figure 2A (Table S2). Through Western blot experiments, we detected the presence of the CDC23 protein in the pull-down samples of the MEG3 sense strand, whereas it was not detected in the antisense strand (Figure 2D). RNA immunoprecipitation assay (RIP) electrophoresis and RT-qPCR results showed that CDC23 binds and significantly enriches MEG3 (Figure 2E,F).

In order to further determine the interaction relationship, we truncated the full length of MEG3 (Figure 2G) and the results showed significant binding of CDC23 at the 425 nt–897 nt position of MEG3 (Figure 2G). The above experimental results demonstrated the authentic binding between MEG3 and the CDC23 protein. This implied that CDC23 may be a novel participant in the regulation of skeletal muscle satellite cell growth and development. Moreover, to investigate whether there were any associations between CDC23 and the cell cycle pathway during the differentiation process, we constructed a protein–protein interaction network to reveal potential interactions (Figure 2C). The analysis results revealed that the protein with the highest number of interactions was CDC23 (Figure 2B), suggesting a close correlation between CDC23 and the enriched genes in the cell cycle pathway. Therefore, we hypothesized that CDC23 may be involved in the regulation of functional processes during skeletal muscle satellite cell differentiation by influencing the cell cycle pathway.

### 2.3. Expression Pattern of CDC23 in Skeletal Muscle

**Figure 3:**
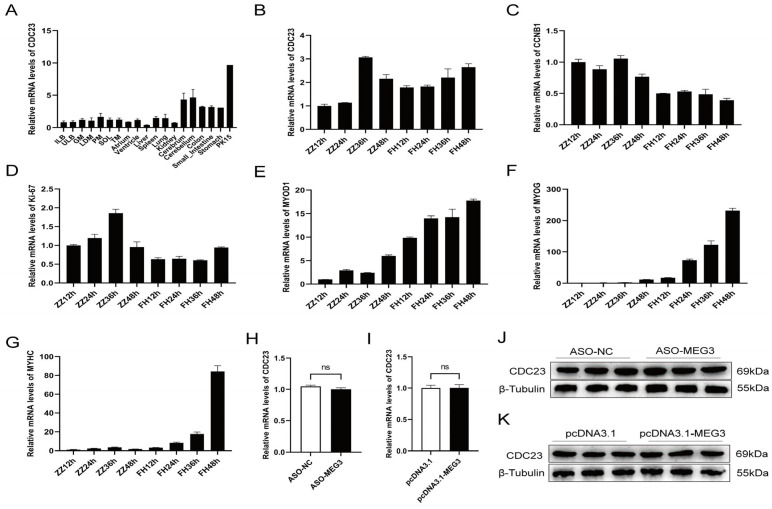
Expression pattern of CDC23 in skeletal muscle. (**A**) CDC23 expression analysis of the different tissues of Rongchang pigs by using RNA-seq data in a public database. (ILB, inner layer of backfat; ULB, upper layer of backfat; GM, gluteus medius muscle; LDM, longissimus dorsi muscle; PM, psoas major; SOL, Soleus; TM, teres major). (**B**–**G**) The mRNA expressions of CDC23, CCNB1, Ki-67, MyoD1, MyoG, and MyHC in different periods of proliferation and differentiation. ZZ represents proliferation and FH represents differentiation. (**H**,**J**) The mRNA and protein expression levels of the CDC23 gene after knockdown of MEG3. (**I**,**K**) The mRNA and protein expression levels of the CDC23 gene after overexpression of MEG3 (ns is considered to be not significant).

Research has shown that CDC23 is a subunit of the Anaphase-Promoting Complex/Cyclosome (APC/C) during the late stage of polyubiquitination, but its function remains unknown. Firstly, we sorted its expression profile with the data from Jin et al. in the GEO public database (GSE162145) [40]. The transcriptomic data demonstrated that CDC23 exhibits varying levels of expression across different tissues in pigs, indicating its broad expression profile (Figure 3A). CDC23 is highly expressed in the cerebrum, cerebellum, and PK15 cell line, but its expression levels are lower in other tissues and organs. Interestingly, compared to other muscles, CDC23 exhibits relatively higher expression in the psoas major muscle. To explore the function of CDC23 in PSCs, we observed the expression pattern of CDC23 in PSCs at different proliferation and differentiation time points (Figure 3B). The results showed that the expression levels of CDC23 were relatively similar between the proliferation and differentiation phases. In the proliferation phase, the peak expression level of CDC23 occurred at 36 h, whereas in the differentiation phase, its expression level increased along the differentiation time points. We also detected the expression level of two proliferation markers and three differentiation markers in different proliferation and differentiation periods. The RT-qPCR results showed that CCNB1 and ki-67 exhibited high levels of expression during the proliferation phase (Figure 3C,D).

Consistent with previous reports, MyoD was expressed at both the proliferative and early differentiation stages, and the expression level during the differentiation phase was several times higher than that during the proliferation phase (Figure 3E). In contrast, MyoG and MyHC are expressed almost exclusively during differentiation (Figure 3F,G). In particular, the mRNA expression level of MyHC increased sharply at the late stage of differentiation. These results indicated that CDC23 may regulate pig growth and development. In order to further validate the relationship between CDC23 and MEG3, rescue experiments were conducted. The results showed that knockdown or overexpression of MEG3 had no significant effect on the mRNA and protein expression levels of the CDC23 gene (Figure 3H–K).

### 2.4. CDC23 Promotes Myoblast Proliferation

**Figure 4:**
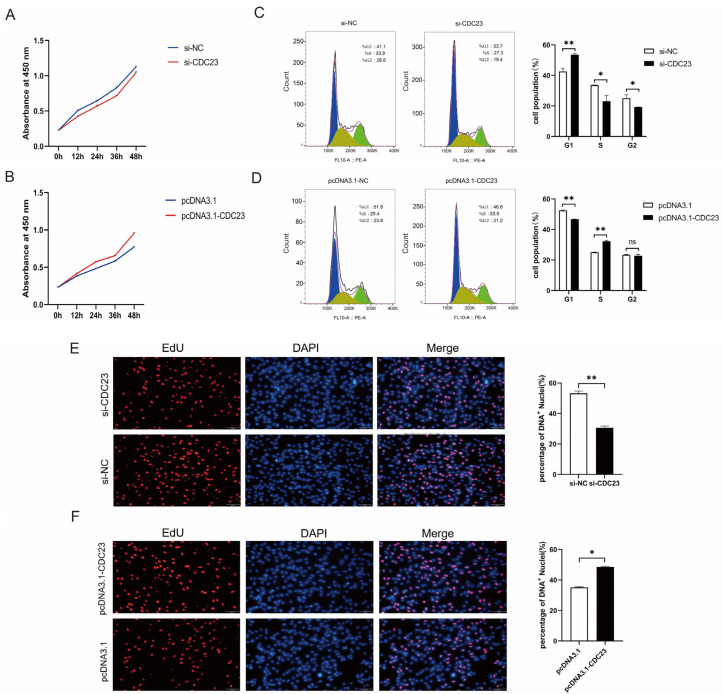
CDC23 promotes myoblast proliferation. (**A**) CCK-8 cell proliferation assay after knockdown of CDC23. (**B**) CCK-8 cell proliferation assay after overexpression of CDC23. (**C**) Flow cytometry analysis after knockdown of CDC23. (**D**) Flow cytometry analysis after overexpression of CDC23. (**E**) EdU staining assays after knockdown of CDC23. (**F**) EdU staining assays after overexpression of CDC23. The S phase of mitosis cells was stained with EdU. The nuclei were stained with DAPI. Scale bar: 50 μm. Error bars represent the mean ± SEM of three biological replicates. Statistical differences between groups were determined with a 2-tailed Student’s *t*-test, and a *p*-value < 0.05 was considered statistically significant (* *p* < 0.05, ** *p* < 0.01). ns is considered to be not significant.

Given that CDC23 was upregulated during myoblast proliferation (Figure 3B), CDC23 could be involved in the regulation of myoblast proliferation. We performed inhibition and overexpression experiments to assess its effect on the proliferation of myoblasts. The CDC23 inhibition/overexpression vectors were constructed and, respectively, transfected into porcine satellite cells cultured in GM. The knockout efficiency of si-CDC23-2 was superior, and it was subsequently employed in the following experiments. The CCK-8 assay showed that the overexpression of CDC23 for 24, 36, or 48 h could dramatically accelerate cellular proliferation (Figure 4B). Inversely, the CDC23 knockdown substantially suppressed the proliferative ability of porcine satellite cells compared with the negative control (Figure 4A). In the 5-ethynyl-2′-deoxyuridine (EdU) staining assays, the overexpression of pcDNA3.1-CDC23 showed higher mitotic activity with an increase in EdU incorporation (Figure 4F). On the contrary, the interference of si-CDC23 showed lower mitotic activity with a decrease in EdU positivity (Figure 4E).

Moreover, flow cytometric analysis revealed a considerable reduction in cell quantity in the G1 phase and a remarkable increase in cell quantity in the S phase after the overexpression of CDC23 (Figure 4D). Conversely, the CDC23 knockdown showed an opposite effect (Figure 4C). These findings validated that CDC23 can promote the proliferation of porcine satellite cells.

### 2.5. CDC23 Positively Regulates Myogenic Differentiation and Myogenin Expression

**Figure 5:**
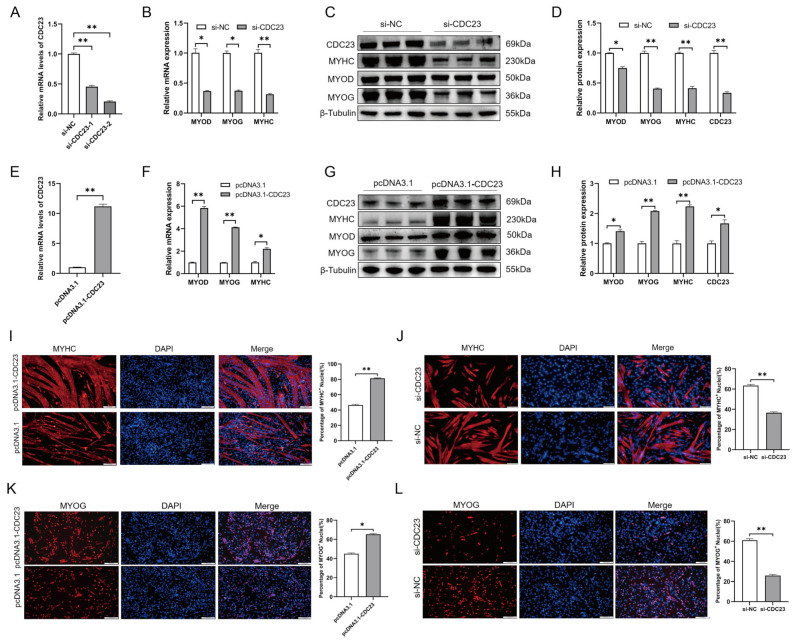
CDC23 overexpression positively modulates myoblast differentiation. (**A**) Efficiency assay of CDC23 interfering RNA. (**B**) The mRNA expressions of MyoD, MyHC, and MyoG were decreased after CDC23 knockdown. (**C**) The protein expressions of MyoD, MyHC, and MyoG were decreased after CDC23 knockdown. (**D**) Quantitative analysis of Western blot results after knockdown of CDC23. (**E**) Efficiency measurement of the CDC23 overexpression vector. (**F**) The mRNA expressions of MyoD, MyHC, and MyoG were decreased after CDC23 overexpression. (**G**) The protein expressions of MyoD, MyHC, and MyoG were decreased after CDC23 overexpression. (**H**) Quantitative analysis of Western blot results after overexpression of CDC23. (**I**) Overexpression of CDC23 increased MyHC-positive porcine satellite cells. (**J**) Knockdown of CDC23 reduced MyHC-positive porcine satellite cells. (**K**) Overexpression of CDC23 increased MyoG-positive porcine satellite cells. (**L**) Knockdown of CDC23 reduced MyoG-positive porcine satellite cells. The scales are all 100 μm. Error bars are the mean ± SEM of three biological replicates. Statistical differences between groups were determined with a 2-tailed Student’s *t*-test, and a *p*-value < 0.05 was considered statistically significant (* *p* < 0.05, ** *p* < 0.01).

The results (shown in Section 2.4) demonstrated that CDC23 is important for myoblasts to be able to withdraw from the cell cycle, a crucial step in myoblast differentiation. In addition, the expression profile of CDC23 indicated its association with myoblast differentiation (Figure 3B). To study the role of CDC23 in PSC differentiation, the overexpression vectors pcDNA3.1-CDC23 and si-CDC23 were transfected into PSCs cultured in DM(Figure 5A,E). We used RT-qPCR and Western blot to test the changes in myogenic marker genes (MyoD, MyoG, and MyHC) after total cellular RNA and proteins were collected. The Western blot results showed that compared with the control group transfected with pcDNA3.1, CDC23 in the overexpression group increased significantly (Figure 5G). In contrast, the si-CDC23 transfection group showed significantly reduced expression of CDC23 (Figure 5C). After overexpression of CDC23, we found that the expression levels of MyoD, myogenin (MyoG), and MyHC, which are differentiation marker genes, were significantly increased compared with the control group (Figure 5F–H). Meanwhile, after transfection of si-CDC23, the expression of the markers all significantly decreased (Figure 5B–D). In addition, the Western blot results revealed a strong correlation between the protein expression levels and mRNA levels of the three myogenic markers following CDC23 knockout and overexpression.

To further investigate the role of CDC23 in myoblast differentiation, we conducted immunofluorescence staining experiments. The results of immunofluorescence staining indicated that overexpression of CDC23 significantly increased the number of MyHC+-positive cells and the size of myotubes, whereas the opposite results were observed after interference (Figure 5I,J). Similar results were observed in the MyoG immunofluorescence assay, where knockdown of CDC23 significantly reduced the number of MyoG+-positive cells, whereas overexpression resulted in the opposite effect (Figure 5K,L). In summary, these results indicated that CDC23 positively regulates the transcription of myogenic factors and differentiation of porcine skeletal muscle satellite cells.

### 2.6. CDC23 May Participate in the Cell Cycle Pathway to Regulate Skeletal Muscle Differentiation

**Figure 6:**
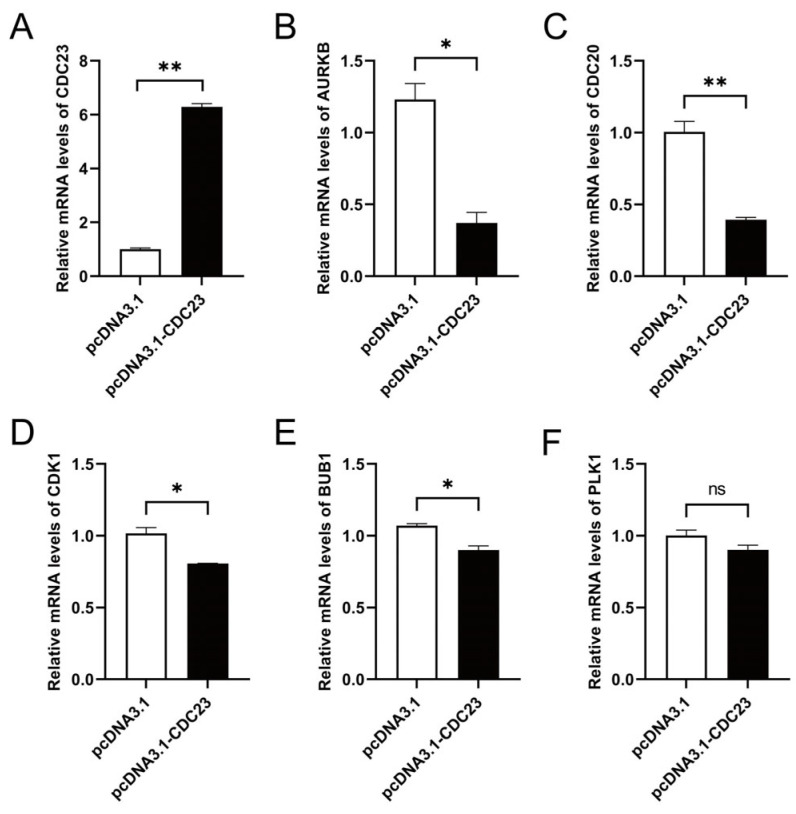
CDC23 negatively regulates cell cycle signaling pathways. (**A**) Efficiency detection of the CDC23 overexpression vector. (**B**–**E**) The mRNA expressions of AURKB, CDC20, CDK1, and BUB1 were significantly decreased after CDC23 overexpression. (**F**) The mRNA expressions of PLK1 showed no change after CDC23 overexpression. Error bars are the mean ± SEM of three biological replicates. Statistical differences between groups were determined with a 2-tailed Student’s *t*-test, and a *p*-value < 0.05 was considered statistically significant (* *p* < 0.05, ** *p* < 0.01). ns is considered to be not significant.

There is a highly interdependent relationship between muscle differentiation and cell cycle regulation. To verify whether CDC23 can affect the cell cycle signaling pathway, we performed pathway marker gene detection. Several genes related to the cell cycle signaling pathway were selected for validation with RT-qPCR. Cell cycle pathway-related genes (AURKB, CDC20, CDK1, and BUB1) were prominently reduced at the mRNA level by CDC23 overexpression (Figure 6A) in skeletal muscle satellite cells (Figure 6B–E). In addition, the expression of PLK1 is not significant, but there is a downward trend with enhanced CDC23 expression (Figure 6F). These data indicated that CDC23 may regulate muscle growth and development by inhibiting the cell cycle pathway.

## 3. Discussion

The proliferation and differentiation of myocytes are crucial processes in skeletal muscle development, and they determine the quality and quantity of meat production in livestock animals. Elucidating the regulatory mechanisms of muscle development can contribute to the improvement of meat quality in animal production, and will provide new insights for identifying therapeutic targets in the treatment of muscular diseases in the future. Therefore, investigating the development of skeletal muscle is of significance. The specific function and signaling mechanism of CDC23 in skeletal muscle cells remained unclear, underpinning the relevance of further research in this area.

Skeletal muscle development is a complex biological process [41] involving not only the proliferation and differentiation of myogenic cells but also the fusion of myotubes to form various types of muscle fibers and the establishment of the functional architecture of skeletal muscle. Our results revealed that CDC23 exhibits widespread expression in various tissues and organs, and its high degree of expression in the PK15 cell line provides additional evidence for its role in cancer. We speculated that CDC23 may be involved in cellular proliferation and migration processes. Similarly, our data demonstrated that the expression level of CDC23 increases over time at different proliferation and differentiation stages, reflecting its close association with the development of skeletal muscle satellite cells. The binding protein CDC23 captured our attention as a partner of MEG3. However, subsequent experiments revealed that knocking down or overexpressing MEG3 had minimal effect on the mRNA and protein expression levels of the CDC23 gene. We speculated that there may exist a complex and unknown regulatory relationship between them, and further exploration will need to be conducted in the future.

Proliferation and differentiation are crucial processes in skeletal muscle development. Cyclin E and CCND1 can bind to cyclin-dependent protein kinases to control cell cycle progression, such as the G1-S and G2-M transition [42,43]. The expression of Ki67 is closely associated with cell proliferation and growth and is commonly used as a proliferation marker in various tumor lesions [44,45]. The results of this study indicated a positive correlation between the expression levels of CDC23 and the genes CCNB1 and Ki67 during skeletal muscle satellite cell proliferation. In addition, the EDU and CCK-8 assays also demonstrated that CDC23 significantly promotes the proliferation of skeletal muscle satellite cells. The results of flow cytometry showed that both gain-of-function and loss-of-function experiments of CDC23 significantly affected the cell cycle progression. Previous reports have indicated that cell proliferation is regulated by cell cycle progression [46]. These findings were consistent with our data and further support the potential of CDC23 in promoting porcine satellite cell proliferation.

Previous studies have indicated that myogenesis differentiation is regulated by a complex network of myogenic transcription factors, such as MyoD, Myf5, MyoG, MRF4, and MyHC [47,48]. MyoD is considered a master regulatory gene for myogenic differentiation [49], whereas MyoG is indispensable for the terminal differentiation of myocytes [48]. Previous studies have shown that MyHC and MyoG can be used as markers of myoblast differentiation [50]. Our data demonstrated a highly consistent trend between the expression levels of differentiation marker genes and CDC23 at both the mRNA and protein levels, regardless of whether CDC23 was knocked out or overexpressed. The results suggested a close association between CDC23 and skeletal muscle satellite cell differentiation. Immunofluorescence experiments indicated that overexpression of CDC23 significantly enhanced cell differentiation compared to the control group, whereas knockdown of CDC23 inhibited cell differentiation. As demonstrated by the significant changes observed in the fluorescence images of MyHC and MyoG, further evidence supported the positive regulatory role of CDC23 in cellular differentiation. In conclusion, CDC23 promotes both proliferation and differentiation in porcine skeletal muscle satellite cells. These results suggested that CDC23 may be involved in regulating the growth and development of skeletal muscle satellite cells through different regulatory factors or pathways. However, the specific regulatory mechanism remains unknown, and further investigation is needed in the future.

The signaling pathways underlying muscle development are complex [51], and currently little is known about the regulatory signals involving CDC23 in skeletal muscle. Cell-cycle arrest is a prerequisite to the differentiation of myoblasts into mature myotubes [52]. MYBL2 regulates cell cycle progression, cell differentiation, and survival [53] by promoting cell cycle progression [54]. CEND1 (cell cycle exit and neuronal differentiation protein 1) plays an important role in neuronal differentiation by modulating cell cycle progression/exit or apoptosis of neuronal progenitors [55]. Abnormal cell cycle progression, such as cell cycle arrest, induces ESC differentiation or apoptosis [56,57]. In our transcriptome analysis results, the cell cycle pathway was significantly downregulated during skeletal muscle satellite cell differentiation. In addition, PPI analysis revealed a close association between CDC23 and enriched genes in the cell cycle pathway. We speculate that CDC23 is involved in the regulation of the cell cycle pathway. However, the specific regulatory sites remain unclear. Research has revealed that the repressive histone mark H3K27me3 regulates myogenic differentiation via the silencing of muscle-specific genes and cell cycle genes [58,59]. MyoD1 is involved in proliferating myoblasts and regulating muscle cell differentiation through the stimulation of cell cycle arrest [60]. DGKZ is a negative regulator of cell cycle progression [61], such that decreased expression of DGKZ (via siRNA) impairs muscle cell differentiation [62]. Furthermore, Msx1 was shown to block cellular differentiation by preventing cell cycle exit [63]. Our current research findings were consistent with previous reports, indicating that CDC23 inhibits cell cycle signaling by downregulating the expression of AURKB, CDC20, CDK1, and BUB1. It is worth noting that CDC23 does not affect PLK1. These results further demonstrated that CDC23 may function as an inhibitory factor in the cell cycle pathway, regulating muscle development. In summary, these observations suggested that CDC23 plays a positive regulatory role in muscle growth and development, possibly through the inhibition of the cell cycle signaling pathway.

**Figure 7:**
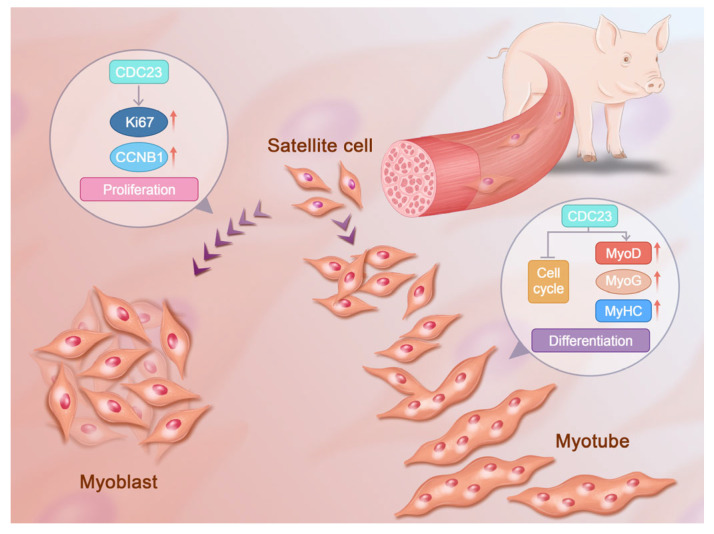
Molecular model of the CDC23 regulation of PSC proliferation and differentiation.

However, the mechanisms of CDC23 to regulate PSC growth and development have not yet been thoroughly elucidated. In addition, the regulatory relationship between CDC23 and the cell cycle pathway could be further validated through the specific activators of the cell cycle pathway. It is necessary to clarify the form of CDC23 that inhibits key sites of the cell cycle pathway. CDC23 may have many other functional roles that need to be explored, and future efforts will be devoted to the detailed analysis of the other diverse functional mechanisms through which CDC23 regulates PSC differentiation. Here, we present a molecular model to elucidate the role of CDC23 in regulating PSC differentiation (Figure 7). This study was the first to identify and report the mechanisms of CDC23 in PSC proliferation and differentiation and may provide some molecular basis for the future research of porcine myogenesis.

## 4. Materials and Methods

### 4.1. Animal and Ethics Statement

The animal used in this study was a 7-day-old, 3.1 kg, male Large White piglet. For porcine satellite cell isolation, the muscles of extremities from the piglets were rapidly pooled, minced, and digested. Using a sterile surgical knife, we cut off various parts of muscles including triceps brachii, biceps femoris, semitendinosus, semimembranosus, and gastrocnemius, and preserved them in PBS containing 1% antibiotic-antimycoti. It is preferable to complete the entire process within 10 min. Please refer to the “Animals and PSCs Isolation” section [15] for the detailed experimental procedures. The PAX7 gene is an important marker for the identification of skeletal muscle satellite cells, and the test results are shown in Figure S1.

Animal care and experimentation procedures in this study were carried out in accordance with the guidelines from Regulation Proclamation No. 5 of the Standing Committee of Hubei People’s Congress. All experimental protocols were approved by the Institutional Animal Care and Use Committee of Huazhong Agricultural University, Wuhan, China (permit HZAUSW2015-0003).

### 4.2. Gene Set Enrichment Analysis of DEGs

Gene Set Enrichment Analysis (GSEA) of the DEGs [15] was conducted using the gseaGO and gseaKEGG functions within the R package ClusterProfiler [64]. The show category number was set as 10. The org.Ss.eg.db package was applied to map the gene identifiers. Dotplots were created using the GseaVis and ggplot2 packages [65]. Gene sets with |NES| > 1 and FDR (padj) < 0.25 were considered to be significant. The database STRING (version 12.0, https://string-db.org/ (accessed on 6 September 2023)) was used to study protein–protein interactions [66].

### 4.3. Cell Culture

PSCs were cultured in a growth medium (GM) containing 76.5% RPMI 1640 (Gibco, Los Angeles, CA, USA, Cat#A10491), 20% FBS (Wenren Biotechnology, Shanghai, China, Cat#FBS-AUS050), 0.5% chicken embryo extract (Gemini, Woodland, CA, USA, Cat#100-163P), 1% GlutaMax (Gibco, Los Angeles, CA, USA, Cat#35050-061), 1% non-essential amino acids (Gibco, Los Angeles, CA, USA, Cat#11140-050), 1% antibiotic-antimycotic (Gibco, Los Angeles, CA, USA, Cat#15240-062), and 2.5 ng/mL human recombinant basic fibroblast growth factor (Gibco, Los Angeles, CA, USA, Cat#13256029). When the PSCs reached 80% confluence, the GM was replaced with a differentiation medium (DM) containing DMEM supplemented with 2.5% horse serum (Gibco, Los Angeles, CA, USA, Cat#26050088). The cells were cultured at 37 °C with 5% CO_2_.

### 4.4. RNA Pull-Down Assay

After linearization of the plasmids, T7 RNA polymerase (Roche, Mannheim, Germany) and biotin RNA labeling mix (Roche, Mannheim, Germany) were used to synthesize transcripts of the MEG3 full-length and mutant fragments. Then, the transcripts were treated with DNase I and EDTA. Proteins were extracted and lysed from the PSCs. In vitro biotinylated RNAs (3 µg) were incubated with the proteins overnight, then the complex was pulled down with streptavidin beads. The beads were washed five times with a wash buffer. Then, the protein complexes associated with beads were analyzed with mass spectrometry and Western blot.

### 4.5. RNA Immunoprecipitation Assay

We performed RNA immunoprecipitation (RIP) assays using an EZ-Magna RIP kit (Millipore, Billerica, MA, USA). Briefly, RIP lysis buffer was used to lyse 107 cells, and the lysates were incubated with 10 μg CDC23 antibody (Abclonal, Wuhan, China, Cat#A6025) at 4 °C overnight. Then, we added the protein A/G beads to pull down the RNA-protein complex. Subsequently, the RNA was purified from the complex and the abundance of MEG3 was detected with RT-qPCR. The 18S rRNA was used as an internal control.

### 4.6. Western Blot

The protein expression levels of the myogenin (MyoG) gene, myogenic differentiation (MyoD), myosin heavy chain (MyHC), and CDC23 in the PSCs were detected by performing immunoblotting. Transfected cells were lysed in RIPA buffer with 1% PMSF and the protein was loaded onto an SDS-PAGE gel and transferred onto a PVDF membrane. Non-specific binding was blocked with 5% non-fat milk in Tris-buffered saline with Tween 20 for 2 h. Then, the proteins were incubated with anti-MyoG (1:1000, Abclonal, Wuhan, China, Cat#A17427), anti-MyoD (1:1000, Proteintech, Wuhan, China, Cat#18943-1-AP), anti-myosin heavy chain (MyHC; 1:3000, Millipore, Darmstadt, Germany, Cat#05-716), CDC23 antibody (1:1000, Abclonal, Wuhan, China, Cat#A6025), and anti-β-tubulin (1:3000, Proteintech, Wuhan, China, Cat#10068-1-AP) at 4 °C overnight. The blots were subsequently incubated with HRP-conjugated secondary antibodies (1:4000), including HRP-labeled goat anti-mouse IgG (Servicebio, Wuhan, China, Cat#GB23301), and HRP-labeled goat anti-rabbit IgG (Servicebio, Wuhan, China, Cat#GB23303). ECL substrates were used to visualize the signals (Beyotime, Shanghai, China, Cat#P0018A). Image J software (version 1.53e) was used to conduct a quantitative analysis of the Western blotting results, according to the gray value of the strip.

### 4.7. RNA Oligonucleotide, Plasmid Construction, and Cell Transfection

The small interfering RNA (siRNA) of CDC23 were purchased from Sangon Biotech (Shanghai, China). The siRNA sequences used are shown below:

CDC23 siRNA-1: GCAGUUGCCUAUCACAAUATT

CDC23 siRNA-2: GGAGUAAAGCUUUACGCUUTT

For the construction of CDC23 overexpression vectors, 2 × Ezmax^®^ Universal CloneMix (Tolobio, Shanghai, China, Cat#24305-02) was used to clone the sequence of CDC23, and then the sequences were inserted into the pcDNA3.1 vector.

For cell transfection, the relevant plasmids or siRNA were used with the jetPRIME^®^ transfection reagent (Polyplus, Illkirch, France, Cat#101000046), as advised by the manufacturer’s protocol.

### 4.8. Reverse Transcription-Quantitative Polymerase Chain Reaction (RT-qPCR)

Total RNA was extracted from the PSCs using a Steady Pure RNA Extraction Kit (Accurate Biology, Changsha, China, Cat#AG21024), according to the manufacturer’s instructions. Then, we used the ABScript||cDNA First-Strand Synthesis Kit (Abclonal, Wuhan, China, Cat#RK20400) to carry out cDNA synthesis for mRNA. The RT-qPCR was carried out on a Bio-Rad PCR System using 2× Universal SYBR Green Fast qPCR Mix (Abclonal, Wuhan, China, Cat#RK21203) and gene-specific primers. The primers are shown in Table S1. As previously described, the 2^−∆∆CT^ [67] method was used to analyze the RT-qPCR data.

### 4.9. CCK-8 Assay

We conducted the experiment based on CCK-8 kit (Abclonal, Wuhan, China, Cat#RM02823) instructions. When the cell density in the 96-well plate reached around 40%, CDC23 knockdown and overexpression were performed, respectively. A total of 10 μL/well of CCK-8 reagent was added at different time periods after transfection: 0 h, 12 h, 24 h, 36 h, and 48 h. Then, culturing was allowed to proceed for 1–4 h. Microplate readers detected the absorbance of different treatment groups at 450 nm and analyzed the proliferation status of the cells.

### 4.10. Flow Cytometry Analysis

Flow cytometry analysis of the cell cycle was performed using a Cell Cycle Assay Kit (Beyotime, Shanghai, China, Cat#C1052). Briefly, the transfected cells were harvested and fixed in 70% ethanol overnight at 4 °C. Then, the cells were rinsed with PBS and centrifuged at 2500 rpm for 5 min. Subsequently, the cells were stained with a pre-prepared propidium iodide (PI) solution, containing RNase A and PI at a volume ratio of 1:9, before incubation in the dark for 30 min. Flow cytometry analysis was performed on a Beckman Coulter FC500 Cytometer (Beckman Coulter, Miami, FL, USA), and the data were processed using FlowJo v10 software.

### 4.11. 5-Ethynyl-20-Deoxyuridine (EdU) Assay

We conducted this experiment according to the instructions for the BeyoClick™ EdU Cell Proliferation Kit with Alexa Fluor 555 (Beyotime, Shanghai, China, Cat#C0075S). When the cell density of the 12-well plate reached 40%, the cells were transfected separately. When the cell density reached 70%, we added EdU reagent to a final concentration of 50 μM for each well, followed by culturing for 1.5–2 h. The cells were then fixed at room temperature with 4% paraformaldehyde solution for 30 min, followed by the addition of 0.5% Triton X-100 and incubation for 10 min for cell permeabilization. Subsequently, we added the pre-prepared Apollo staining solution and incubated the solution for a further 30 min at room temperature, protected from light. The nucleus was stained with 4, 6-diamidino-2-phenylindole (DAPI). Finally, the staining results were observed using a fluorescence microscope, and three visual fields of view were randomly selected for photographing. The changes in the number of EDU-positive cells were compared between the experimental group and the control group.

### 4.12. Immunofluorescence Staining

The cells were fixed in 4% paraformaldehyde for 15 min and then permeabilized in 0.3% Triton X-100 for 15 min. Subsequently, the cells were blocked with blocking solution (3% bovine serum albumin (BSA), 0.3% TritonX-100, 10% FBS complemented with PBS) for 2 h. Then, Anti-MyHC (1:1000; Millipore, Billerica, MA, USA) or anti-MyoG (1:500; Abclonal, Wuhan, China, Cat#A17427) were added and the solution was incubated overnight at 4 °C. After that, the cells were stained with CoraLite594-conjugated Goat Anti-Rabbit IgG (H + L) (Proteintech, Wuhan, China, Cat#SA00013-4) for 1 h. The cell nuclei were stained using DAPI (Servicebio, Wuhan, China, Cat#G1012-10ML) solution in darkness for 10 min. Images from three randomly selected fields were obtained with a Leica SP8 confocal microscope and processed with Image J software (version 1.53e).

### 4.13. Statistical Analysis

Generally, the results are presented as the means ± standard error of the mean (SEM). Statistical differences between groups were determined with a 2-tailed Student’s *t*-test, and a *p*-value < 0.05 was considered statistically significant (* *p* < 0.05, ** *p* < 0.01, *** *p* < 0.001).

## Data Availability

The data presented in this study are available upon request from the corresponding author.

## Supplementary Materials

The following supporting information can be downloaded at: https://www.mdpi.com/article/10.3390/ijms25073664/s1.
