# Supplementary figures and images for: Molecular Regulation of Porcine Skeletal Muscle Development: Insights from Research on CDC23 Expression and Function
Source: Int J Mol Sci. 2024 Mar 25;25(7):3664. doi: 10.3390/ijms25073664 (PMC11011816; doi:10.3390/ijms25073664)

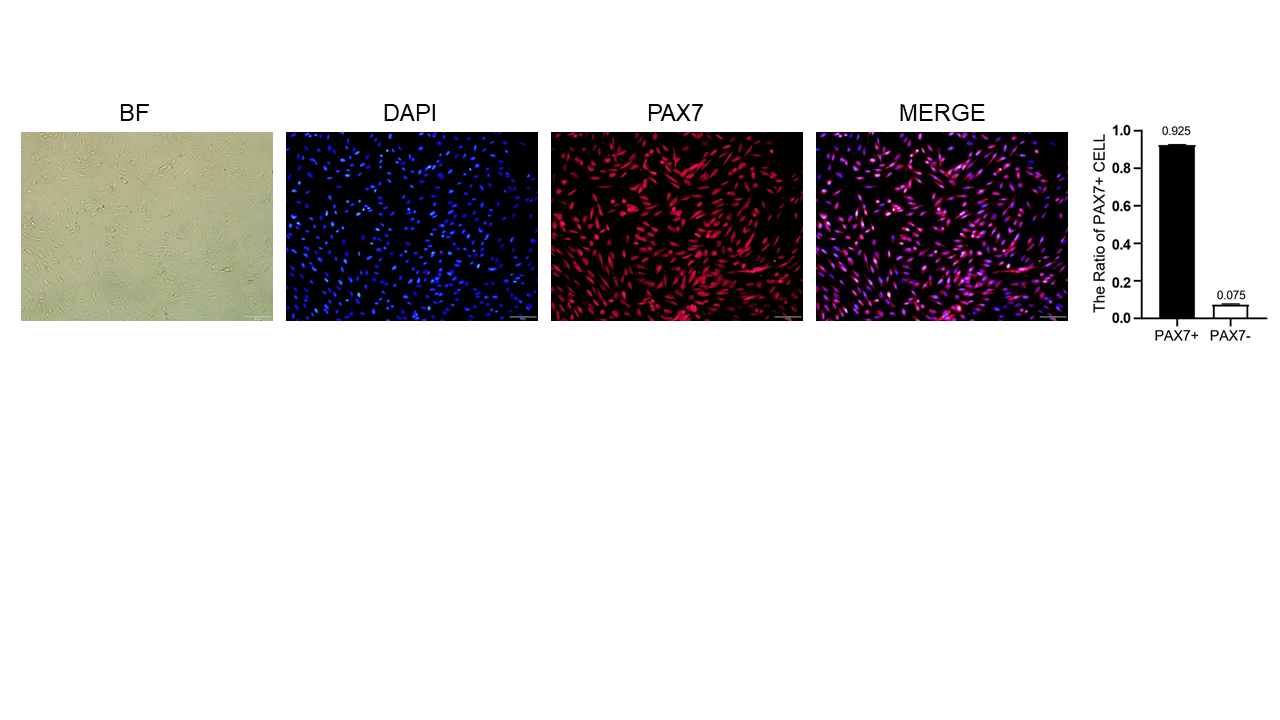

Supplement: Supplementary file 1 [file ijms-25-03664-s001.zip › Supplementary Materials/Figure S1_Pax7+.tif]
